# A novel bone graft technique combined with plating for aseptic recalcitrant long bone nonunion

**DOI:** 10.1186/s12891-022-05830-8

**Published:** 2022-09-20

**Authors:** Yuxuan Jiang, Xiaolong Wang, Wei Huang, Yangjun Zhu, Kun Zhang, Dongxu Feng

**Affiliations:** Department of Orthopaedic Trauma, Hong Hui Hospital, Xi’an Jiaotong University School of Medicine, Youyi Road, Xi’an, Shaanxi Province 710054 China

**Keywords:** Bone-forming channel technique, Nonunion, Bone graft, Locking compression plate, Autogenous structural iliac bone

## Abstract

**Background:**

To evaluate the outcomes and efficacy of a new technique of autogenous iliac crest bone grafting combined with locking compression plate (LCP) vertical fixation for aseptic recalcitrant long bone nonunion.

**Methods:**

From July 2010 to September 2020, 36 aseptic recalcitrant long bone nonunions were treated with a bone-forming channel technique and internal LCP fixation. All the patients had received one or more failed treatments. The injury mechanism, nonunion type and duration, and prior treatments were recorded pre-operation. The routine treatment process included nonunion area exposure, previous implant removal, sclerotic bone debridement, LCP fixation, bone-forming channel creation, and iliac bone grafting, and a second LCP fixation when required. At follow-up, X-ray images were obtained to assess bone healing and implant failure. Visual analog scale (VAS), fracture site stability, limb function, activity, muscle strength, limb length, and complications were recorded.

**Results:**

A total of 34 patients (24 males and 10 females) were finally enrolled, with a mean age of 49.8 ± 12.3 years. At a mean follow-up of 35.6 ± 22.0 months, 32 patients displayed bone union, with a healing rate of 94.1% and mean union time of 6.8 ± 2.4 months. The VAS score was 0.7 ± 1 at the final follow-up. The functional results showed that 19 patients were excellent, 11 patients were good, 2 patients were poor, and 2 patients did not heal.

**Conclusion:**

Bone-forming channel technique combined with LCP vertical fixation is an excellent option to treat recalcitrant long bone nonunion.

**Level of evidence:**

Therapeutic Level IV.

## Background

Although conservative treatment and surgical techniques have greatly improved, the incidence of nonunion remains at close to 5–10% [1]. The incidence rate of nonunion in most long bones is > 10% [2]. Bone nonunion that meets the diagnosis displays poor self-healing and normally requires therapeutic intervention. In recent years, many treatment methods of bone nonunion have been reported, including plate and intramedullary nail with bone graft, external fixation, biological stimulation, and pulsed ultrasound [3–7]. The prognosis for patients who received these treatments was satisfactory. Therefore, the majority of nonunions can be effectively managed with these techniques.

Clinical orthopedic doctors still encounter patients with recalcitrant long bone nonunion. This is frequently accompanied by unstable soft tissue coverage, poor osteogenesis, and severe disability. Long-term nonunion leads to patients displaying depression, anxiety, reduced psychological resilience, and other psychological traumata. Repeated treatment also represents a huge economic burden to society and the patients’ families [8, 9]. Therefore, it is necessary to ensure the early healing of nonunion.

The authors’ previous research reported that the nonunion ends were determined as consisting of one osteogenesis deactivation zone (scar tissue and hardened sequestrum) and two osteogenesis activation zones (normal porosis tissue). The authors used a new bone graft technique termed the bone-forming channel technique, which spans the osteogenesis deactivation zone to bridge the osteogenesis activation zones [10]. Combined with this technology, this study adopted a new surgical strategy and achieved good results in clinical treatment [11–13]. In the present study, the authors retrospectively analyzed the patients with recalcitrant nonunion to whom this surgical method was applied, with the belief that this surgical strategy can have a good therapeutic effect on such patients, and to provide some data for clinical treatment.

## Methods

### Ethics approval

The study was approved by the ethics committee of our hospital (No. 202205008). Written informed consent was obtained from all parents and/or guardians.

### Study population

This study retrospectively examined adult patients diagnosed with recalcitrant long bone nonunion who had received at least one failed treatment and had a history of more than 10 years of nonunion in Xi’an Honghui hospital from July 2010 to September 2020. Inclusion criteria were: (1) Patient met diagnostic standards of nonunion: a minimum of 9 months from the last operation and had no further signs of healing in the previous 3 months; and an absence of bridging callus for at least three out of four cortices on plain radiographs. (2) Patients had clinical symptoms such as dysfunction, pain, deformity, and stiffness of adjacent joints requiring surgical intervention. (3) There was no obvious infection on clinical, hematological, or radiological examinations. (4) The results of intraoperative bacterial culture were negative. (5) Patient received locking compression plate (LCP) fixation combined with bone-forming channel technology. (6) The follow-up was longer than 12 months. Exclusion criteria were: (1) Infectious nonunion. (2) Multiple fractures of the same bone. (3) Pathological fracture.

### Surgical technique

The treatment goal was to correct deformities, with strong internal fixation and effective bone grafting to create an optimum mechanical and biological environment for bone healing. Following general anesthesia, patients were normally maintained in the supine position, and the affected limb and contralateral iliac area were routinely disinfected and covered with sterile surgical towels. The surgical incision was normally performed at the site of the original surgical incision. With the Judet decortication [14], the fracture nonunion site was exposed and the surrounding soft tissue and periosteum were protected as much as possible. The original internal fixation was removed, the wound was completely debrided, and all scar tissue, the entire pseudocapsule, and hardened sequestrum were removed. The surrounding tissues of the nonunion were sent for bacterial culture to preclude a deep indolent infection. When signs of low-grade infection were found during the operation, the operation method was changed. The medullary canal was opened with a drill and reset with the correct bone alignment. Subsequently, an LCP was placed on the side of the long bone for compression fixation to ensure that a minimum of three screws at the distal and proximal ends were fixed through six layers of cortical bone.

**Fig. 1:**
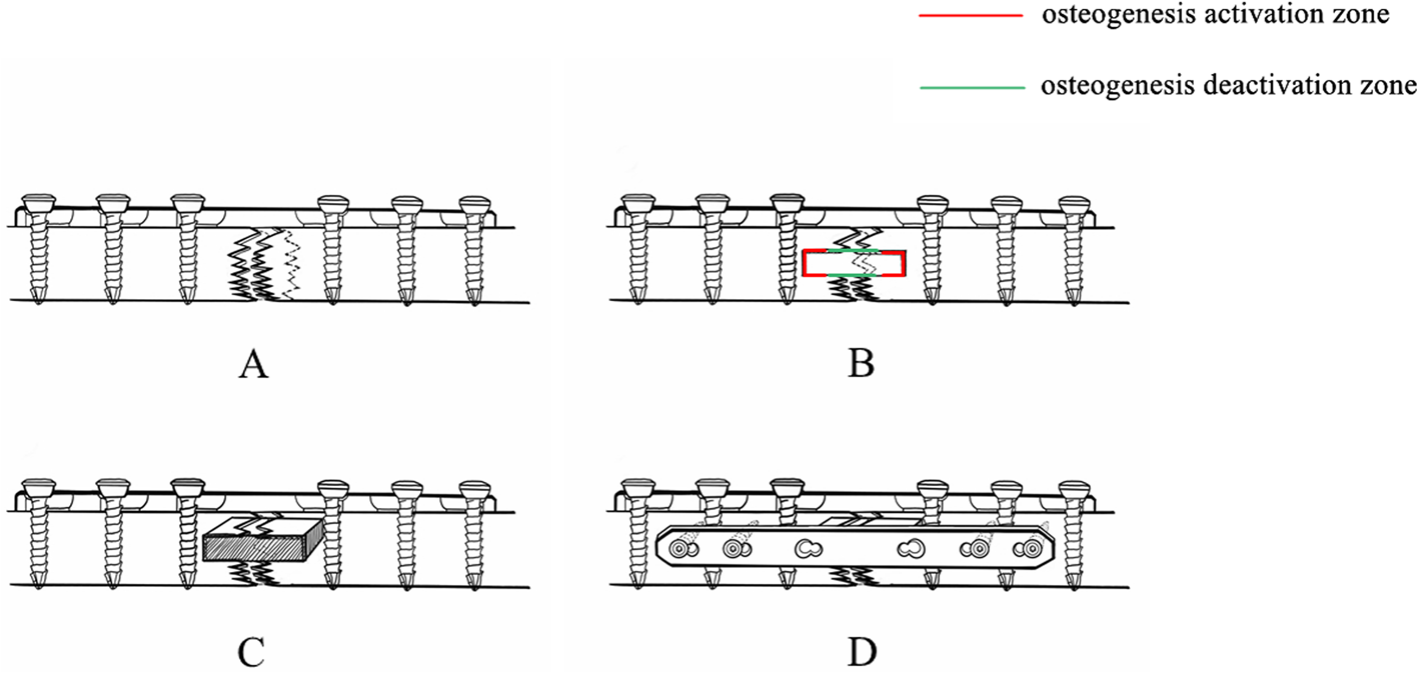
Procedure of bone-forming channel technology combined with LCP. **a** An LCP was placed on the lateral side of the long bone for compression fixation. **b** The proximal and distal areas of the bone nonunion were slotted as preparation for bone grafting. **c** A whole piece of iliac bone of a preset size was placed into the slot. **d** If there was micro movement, a second LCP was placed at the bone graft site

Slotting was subsequently created at the fracture ends on both sides. The slotting should be sufficient to achieve normal bone with fresh punctate blood exudation (paprika sign) [15]. The size and length of the opened bone groove was measured. Structural autologous iliac bone of appropriate size according to the measured bone groove size was obtained. The muscles and periosteum on the obtained iliac bone were completely removed, and the iliac bone was shaped into a long block and planted into the prepared slot. This bone grafting method is termed the bone-forming channel technique (Fig. 1). Excess iliac bone strips were implanted into the gap and around the nonunion to ensure that the bone graft was in full contact with the nonunion site and the structural iliac bone was stable. Subsequently, a second LCP was fixed in front of the bone graft if micro movement occurred at the two nonunion ends, ensuring at least two screws with four cortical fixations on each side.

### Postoperative management

Antibiotic use was routinely initiated 24 h after the operation and the drainage tube was removed after 48 h. On the second day after the operation, active and passive functional exercises of limb joints began under the rehabilitation therapist. External fixation after the operation was not necessary, and early functional exercise can promote functional recovery. As the pain and swelling subsided, the exercise intensity and frequency were increased appropriately, and weight-bearing exercise could be initiated after fracture healing.

### Postoperative follow-up and evaluation

From the third day following surgery, standard anteroposterior and lateral X-ray images were obtained every 4 weeks. Fracture union was indicated by (1) the presence of solid bony bridging on at least three cortical sides; (2) absence of the fracture line; and (3) the patient being able to maintain a full load on the affected lower limb for > 10 min without pain. X-ray examinations were performed to assess the status of the union, internal fixation, and fracture angulation among others.

The clinical function evaluation included the visual analog scale (VAS), fracture site stability, limb function and activity, and muscle strength and length. Intraoperative and postoperative complications were recorded.

In this study, the functional results criteria were based on [10] as follows: (1) excellent: no infection, bone angulation deformity < 7°, and limb shortening < 2.5 cm; (2) Good: combined with one of the following: infection, angulation > 7°, or limb shortening > 2.5 cm; (3) Poor: combined with two or more of the following: infection, angulation > 7°, limb shortening > 2.5 cm, or other conditions affecting functions; (4) Fracture nonunion.

### Statistical analysis

Categorical data are presented as number and percentage, while continuous quantitative data are presented as mean ± standard deviation. Descriptive statistical analyses were performed using SPSS (version 25; IBM Corporation, Armonk, NY, USA).

## Results

In this retrospective study, 36 patients met the criteria, of whom 2 patients were lost to follow-up, such that finally 34 patients (24 males and 10 females) were enrolled, with a mean age of 49.8 ± 12.3 years (range, 29–75 years). The demographic and clinical data are shown in Table 1.

**Table 1:** Demographic and clinical data

Patient	Sex	Comorbid conditions	Smoking	Mechanism of injury	Time since injury (years)	Bone affected by nonunion	Prior treatments	Number of prior treatments	Type of nonunion
1	M	No	No	Motorcycle accidents (open wound)	12	Tibia	D + P; BG; RIF	3	Synovial pseudarthrosis
2	F	No	No	Tumbling	16	Ulna and radius	P;RIF	2	Synovial pseudarthrosis
3	M	No	No	High fall	17	Supracondylar of femur	P	1	Atrophic
4	M	No	No	Crashing	13	Humeral shaft	C; P + BG	2	Synovial pseudarthrosis
5	M	No	No	Heavy pound	11	Humeral shaft	IN	1	Hypertrophic
6	M	No	No	Tumbling	13	Clavicle	Kirschner wires	1	Atrophic
7	M	No	No	Crashing	12	Humeral shaft	P; IN+BG	2	Atrophic
8	M	No	No	Motorcycle accidents	11	Supracondylar humerus	P	1	Atrophic
9	M	No	No	High fall	11	Proximal humerus	P	1	Synovial pseudarthrosis
10	F	CAD	No	Tumbling	10	Humeral shaft	P	1	Synovial pseudarthrosis
11	F	Hypertension grade II	No	Tumbling	12	Tibia	P + BG	1	Hypertrophic
12	M	No	No	Machine	10	Humeral shaft	P	1	Synovial pseudarthrosis
13	F	No	No	Machine	15	Ulna	C; P + BG	2	Atrophic
14	M	No	No	Motorcycle accidents	20	Humeral shaft	P; BG + P	2	Hypertrophic
15	M	No	Yes.40 Years	Tumbling	31	Humeral shaft	P; P + BG	2	Synovial pseudarthrosis
16	M	No	No	Tumbling	13	Tibia	P; P + BG	2	Atrophic
17	M	No	No	Heavy pound	13	Femoral shaft	P; P + BG; D	3	Atrophic
18	M	No	No	Machine (open wound)	27	Humeral shaft	P; P + BG; P	3	Hypertrophic
19	M	No	Yes.40 Years	Heavy pound	19	Humeral shaft	C; P + BG	2	Synovial pseudarthrosis
20	M	No	No	Tumbling	12	Femoral shaft	IN; IN+BG	2	Hypertrophic
21	F	No	No	Crashing	23	Femoral shaft	P; EF	2	Hypertrophic
22	M	Hypertension grade III	Yes.20 Years	Tumbling	11	Femoral shaft	P; P	2	Hypertrophic
23	M	No	No	Motorcycle accidents	10	Femoral shaft	IN	1	Hypertrophic
24	M	No	No	Crashing (open wound)	10	Femoral shaft	EF; P + BG	2	Hypertrophic
25	F	No	No	Tumbling	12	Ulna	P	1	Synovial pseudarthrosis
26	M	Arrhythmia	No	Crashing (open wound)	30	Tibia	P + BG; D + RIF + C	2	Synovial pseudarthrosis
27	F	No	No	Tumbling	10	Tibia	P + BG; EF + BG;	2	Synovial pseudarthrosis
28	M	No	No	Tumbling	37	Supracondylar humerus	Cast	1	Synovial pseudarthrosis
29	M	No	No	Motorcycle accidents	13	Femoral shaft	P; P + BG; P + BG	3	Hypertrophic
30	M	CAD	No	Heavy pound	14	Femoral shaft	P; P + BG	2	Hypertrophic
31	F	No	No	Tumbling	10	Tibiofibula	P; D + VSD; D + FT; RIF + EF; P + BG;	5	Synovial pseudarthrosis
32	M	No	No	Tumbling	15	Ulna	Wires	1	Synovial pseudarthrosis
33	F	Type II diabetes	No	Tumbling	11	Femoral shaft	P	1	Atrophic
34	F	No	No	Tumbling	16	Femoral shaft	P; P	2	Atrophic

*M* Male, *F* Female, *CAD* coronary artery disease, *P* Plate, *D* Debridement, *C* Cast, *BG* Bone graft, *EF* External fixator, *FT* Flap transplantation, *IN* Intramedullary nail, *RIF* Removing internal fixator, *VSD* Vacuum sealing drainage

**Fig. 2:**
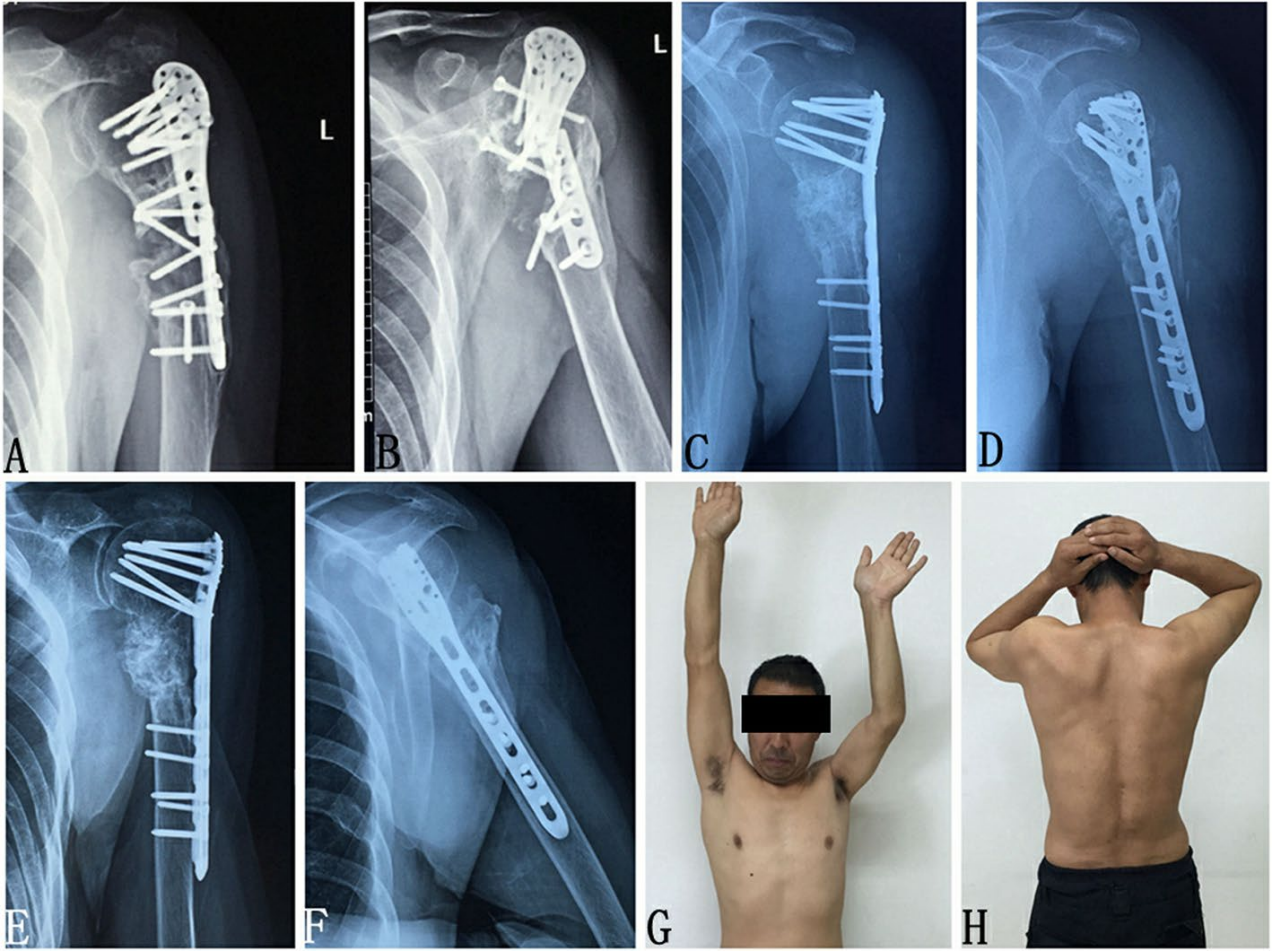
Patient 9, who fell from a height of 4 m 11 years earlier, resulting in ‘left proximal humerus fracture and lumbar fracture’, received surgical treatment in the local hospital. At 8 years ago, the left shoulder had gradually become painful and weak, with limited movement of the left shoulder joint, which was untreated. **a**, **b** Preoperative X-ray shows typical synovial pseudoarticular bone nonunion and broken internal fixation; **c**, **d** Postoperative X-ray shows that the proximal end of the left humerus is well aligned and the bone graft is sufficient; **e**, **f** X-ray at 6 months after the operation shows healed fracture; **g**, **h** Although the left shoulder joint is slightly limited in movement and rotation, the patient is satisfied with the treatment results

**Fig. 3:**
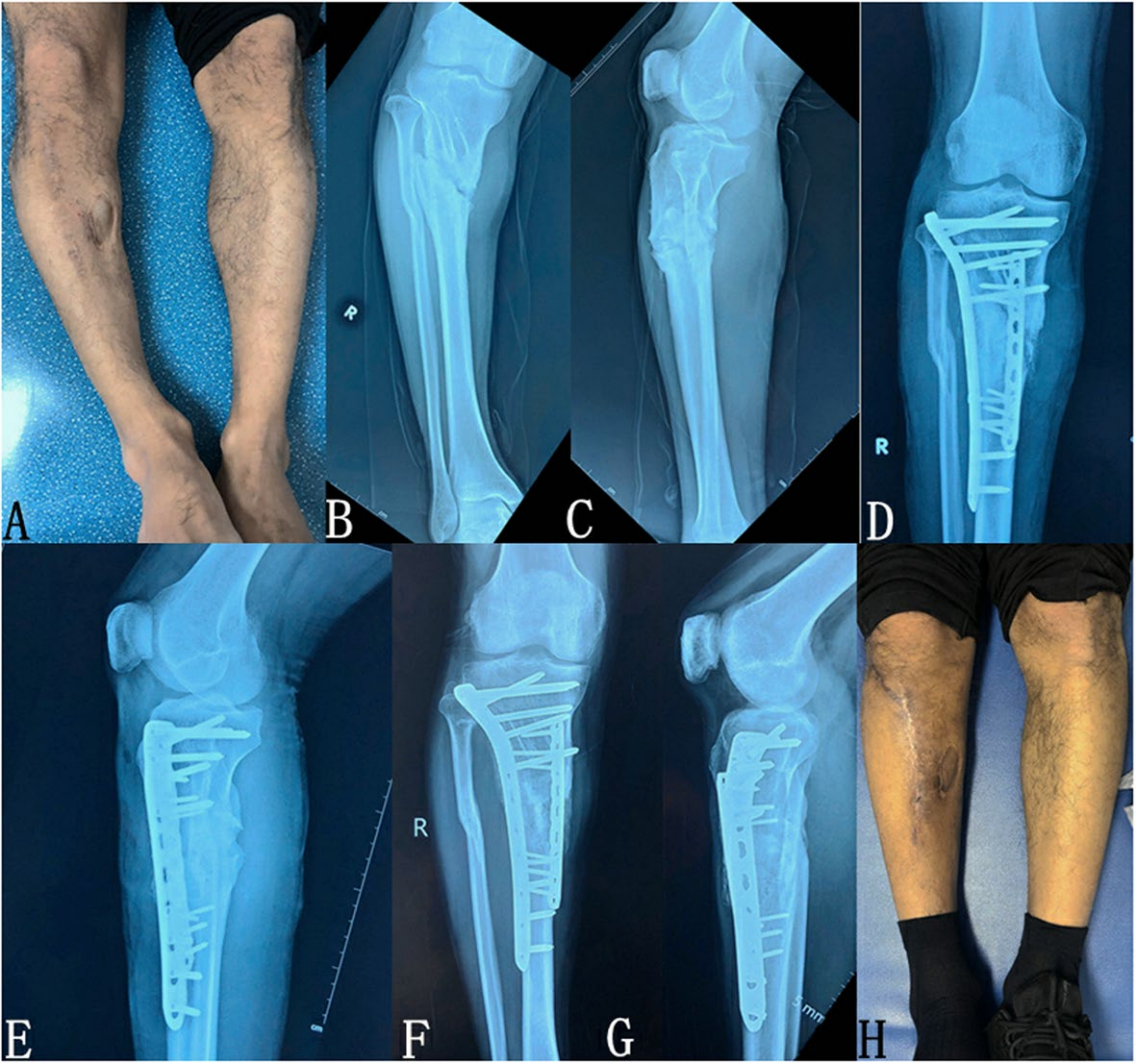
Patient 1, who was injured in a motorcycle accident 12 years earlier, which had caused an open fracture of the right tibia and fibula, received ‘debridement and internal fixation of the open fracture of the right tibia and fibula’ in the local hospital. It was found that the bone healing ability was poor 2 months after the injury, and the patient was thus given iliac bone grafting again. It was found that the tibia was not healed and the plate was broken 5 months after the injury, thus the internal fixation was removed in the local hospital. Subsequently, there was right tibial nonunion, limb deformity, and knee pain. **a** Photo of the affected limb shows varus deformity of the right leg; **b**, **c** Preoperative X-ray shows typical synovial pseudoarticular nonunion with obvious broken end space, accompanied by a poor force line of the right tibia and degenerative changes of the knee joint; **d**, **e** Postoperative X-ray shows that the tibial force line is corrected, the internal fixation position is appropriate, and the bone graft is satisfactory; **f**, **g** X-ray examination 13 months after the operation shows satisfactory tibial healing and (**h**) the appearance of the lower leg has improved

There were three patients with ulnar nonunion, one patient with ulnar and radius nonunion, nine patients with humeral shaft nonunion, one patient with proximal humerus nonunion (Fig. 2), two patients with supracondylar humerus nonunion, ten patients with femoral shaft nonunion, one patient with supracondylar femoral nonunion, one patient with tibiofibular nonunion, five patients with tibial nonunion (Fig. 3), and one patient with clavicular nonunion. A total of 15 patients sustained an injury from tumbling, 5 from motorcycle accidents, 5 from crashes, 4 from a heavy object, 2 from a high fall, and 3 from a machine, and 4 patients had open wounds (Table 1). Among the classification of nonunion, 11 displayed hypertrophic nonunion, 9 an atrophic nonunion, and 14 synovial pseudarthrosis. All patients received had received one or more regular treatments, and the mean number of previous treatments was 1.82 ± 0.87 times (range, 1–5 times). The mean duration since the first treatment was 14.1 ± 7.7 years (range, 5–37 years).

Patients were followed-up for a mean of 35.6 ± 22.0 months (range, 13–78 months). In total, 32 patients displayed union after 6.8 ± 2.4 months (range, 5–18 months), while 2 patients still had nonunion, a healing rate of 94.1%. The VAS score was 0.7 ± 1 at the final follow-up. The functional results showed that 19 patients were excellent, 11 patients were good, 2 patients were poor, and 2 patients did not heal with the implants intact. Five patients had a > 10° coronal angular deformity, four patients had bone shortening of ≥2 cm, two patients had peripheral nerve injury, and one patient had mild pain at the bone donor site. There was no plate breakage or loosening or incision site infection. The patients’ details of prior treatments and functional results of follow-up are presented in Tables 1 and 2.

**Table 2:** Postoperative outcomes

Patient	Number of Plates	Union time (months)	Follow-up period (months)	VAS	Functional results
1	2	6	13	0	Excellent
2	2	7	19	1	Excellent
3	1	6	15	1	Good, angular deformity 15°
4	2	7	18	0	Excellent
5	1	5	14	0	Excellent
6	1	6	21	0	Good, angular deformity >7°
7	1	–	23	3	Did not heal
8	1	5	13	1	Good, angular deformity >7°
9	1	6	19	1	Excellent
10	1	5	15	0	Good, angular deformity >7°
11	2	7	19	0	Excellent
12	1	5	43	1	Good, angular deformity >7°
13	1	5	42	0	Excellent
14	2	6	19	0	Good, angular deformity >7°
15	2	6	45	1	Excellent
16	2	8	21	1	Good, angular deformity >7°
17	2	7	24	1	Good, angular deformity >7°
18	2	5	14	3	Good, radial nerve palsy
19	2	7	27	2	poor, ulnar never palsy, >2.5 cm shorter, angular deformity >7°
20	1	6	42	0	Excellent
21	1	8	15	1	Good, >2.5 cm shorter
22	2	7	27	0	Excellent
23	2	6	29	0	Good, angular deformity >7°
24	2	6	41	0	Excellent
25	1	7	78	0	Excellent
26	1	6	68	0	Excellent
27	2	6	76	0	Excellent
28	1	7	76	1	Excellent
29	2	7	69	0	Poor, 3 cm shorter, mild pain at bone donor site
30	1	12	24	0	Excellent, 1 cm shorter, delayed union
31	1	–	60	4	Did not heal
32	1	18	50	0	Excellent, delayed union
33	2	7	76	0	Excellent
34	2	7	54	1	Excellent

## Discussion

There are many risk factors for bone nonunion, including age, smoking, type II diabetes, energy of injury, severity of soft tissue injury, infection, degree of mechanical stability, inherent degree of vascularization around the fracture, and numerous medications [16, 17]. Some studies have shown that the incidence of nonunion is higher in younger subjects, possibly because high-energy injuries usually occur in younger patients [18, 19]. In this study, nine patients had humeral shaft nonunion and ten patients had femoral shaft nonunion. These two fracture sites are normally caused by a high-energy injury. In studying the injury mechanism, > 50% of the patients had suffered a high-energy injury. Therefore, the energy at the time of injury determines the probability of fracture healing.

At present, there is no unified standard approach for bone nonunion fixation, which is mainly determined according to the bone nonunion location, soft tissue conditions, general condition, and infection [20]. External fixation is mostly used in infected nonunion or patients with poor soft tissue [4, 21]. It has the advantages of providing stimulation to the nonunion site percutaneously and simultaneously controls juxta-articular or ‘emmental’ bone fragments, facilitating staged complex bone and soft tissue reconstruction. Intramedullary nailing is most often used to treat fresh femoral and tibial shaft fractures. Some studies have confirmed that intramedullary nailing is effective in treating aseptic long bone nonunion [20, 22]. It has the advantage of minimal exposure of the nonunion end, which may improve bone union and early weight-bearing [23]. However, both external fixation and intramedullary nailing cannot control rotation, and do not allow auto bone grafting. Moreover, because each patient in the present study had suffered a long period of nonunion, most of them required complete synovial pseudarthrosis debridement and deformities correction during surgery, thus external fixation and intramedullary nails may not have been suitable for the patients in the present study.

In the present study, each recalcitrant nonunion had a nonunion duration longer than 10 years and had received one or more failed previous treatments, and the treatment was challenging for surgeons. Therefore, whether the bone had the ability to heal required consideration, as did whether the factors resulting in nonunion could be reversed by surgery. Due to sufficient stability, fracture ends compression, deformities correction, and bone graft permission, LCP combined with auto bone grafting has been successfully reported for the treatment of nonunion by some authors, with a bone healing rate of up to 100% [24–27]. A detailed preoperative examination and intraoperative observation was conducted in the present study to identify those patients who displayed aseptic bone nonunion. LCP and bone grafting were applied in these patients. This method was not used for those suspected of low-grade infectious nonunion [28]. A positive outcome of this study was that LCP fixation ensured good alignment of nonunion ends after debridement of deformities or synovial pseudarthrosis. In the final follow-up, five patients displayed a coronal angle deformity > 10°, there was no obvious rotation deformity, and the deformity did not affect the function. Another advantage of this study was that the bone-forming channel technique ensured that the autogenous structural iliac bone extended over the osteogenesis deactivation zone and bridged the osteogenesis activation zones, maximizing the osteoinduction and osteoconduction effects of the bone graft, resulting in bone healing. Furthermore, this technology had been successfully used in previous studies, and achieved a satisfactory outcome with a bone union rate of 100% [10–13]. To the best of our knowledge, this is the first time this technique has been applied to treat recalcitrant nonunion. Moreover, some patients were fixed by two LCPs, with the second LCP fixation in front of the bone graft to potentially avoid bone graft migration, thereby improving the bone union rate. Additionally, two LCPs ensured an absolutely stable mechanical environment of the nonunion ends, such that patients might start early functional exercise without an external brace, resulting in good limb function. At the final follow-up, 19 patients had excellent functional results and 11 patients had good functional results, and all patients with bone healing could return to work.

In this study, the bone healing rate was 94.1%, with non-healing in only two patients, of whom one had previously received five failed interventions. This clearly demonstrated the necessity to apply effective treatment measures the first time when presented with nonunion. Jiang et al. treated five patients with refractory postoperative diaphyseal femur fracture nonunion. All the patients had been previously treated with all the standard surgical procedures for fracture nonunion [27]. All the patients achieved bone union following a combination of double LCP plating and an autologous fibula graft. However, the case numbers of this study were small, and fibular harvest may have caused severe complications. Mittal et al. reported 12 patients with recalcitrant nonunion who were treated with double LCP plate fixation combined with decorticated and cancellous bone graft, which was similar to our surgical strategy [29]. All the patients healed within a mean follow-up of 14 months. This study highlighted the considerable effectiveness of osteoperiosteal decortication in the treatment of fracture nonunion and that the LCP provides fixation without negative effects on periosteal vascularization.

In the present study, the method of using an iliac crest bone graft (ICBG) was improved. As early as 1956, Nicoll proposed an ICBG method termed ‘cancellous insert grafts’ to treat patients with long bone nonunion [30]. The classic method was to use a solid block of cancellous iliac bone to bridge the gap in long bones, and in some cases, cortical iliac bone was used as a lid to cover the cancellous grafts. This method was applied in 27 cases, and union was achieved in all of them in a mean time of 14 weeks. In recent studies, the ICBG normally involved splitting of the iliac bone grafts into longitudinal strips followed by implantation around the fracture site [31, 32]. Although these methods are effective, the bone-forming channel technique further improves the biological environment at the bone graft site. Our method with slotting at the proximal and distal regions of the fracture and implantation of the entire structural iliac bone block with cortex and cancellous bone into the slotted area ensures a tight and accurate fit. The combination of Judet decortication and double LCP fixation maximized the fracture union rates.

The present study was limited by its small number of cases and the lack of a control group. This study did not compare its surgical strategy with other treatments. Because this study was retrospective, the fracture healing time could only be judged according to the results of the patient review, which may have been inaccurate. Despite the limitations of the study, the results demonstrated that this novel technique of autologous iliac bone grafting has a good healing rate in patients with recalcitrant bone nonunion. In future research, our sample library will be further expanded, which will provide greater evidence, bringing hope to patients with recalcitrant bone nonunion and reducing the burden of this disease on society and patients’ families.

## Conclusions

This study recommends its described surgical strategy for aseptic recalcitrant long bone nonunion. Judet decortication preserved the blood supply of the fractured ends, double LCP provided mechanical stability, and the bone-forming channel technique provided a favorable biological environment. This method can improve the fracture union rates and rescue many patients with aseptic recalcitrant long bone nonunion. Although the outcome of some patients was not optimal, fracture healing still greatly improved the situation for patients with long-term bone nonunion.

## Data Availability

The datasets used/or analyzed during the current study are available from the corresponding author on a reasonable request.

## Abbreviations

VAS: Visual analog scale
LCP: Locking compression plate
ICBG: Iliac crest bone graft
